# Types and Characteristics of Stress Coping in Women Undergoing Infertility Treatment in Korea

**DOI:** 10.3390/ijerph20032648

**Published:** 2023-02-01

**Authors:** Yumi Choi, So-Hyun Moon

**Affiliations:** 1 College of Nursing, Chungnam National University, Munhwa-ro 266, Jung-gu, Daejeon 35015, Republic of Korea; 2 Department of Nursing, College of Medicine, Chosun University, Pilmundae-ro 309, Dong-gu, Gwangju 61452, Republic of Korea

**Keywords:** infertility, female, stress, psychological, cluster analysis

## Abstract

The purpose of this study was to identify the characteristics and predictors of types of stress coping in women undergoing infertility treatment. The cross-sectional study included 120 women who were receiving infertility treatment at infertility hospitals. Self-report questionnaires were used to measure. K-means cluster analysis and multinomial logistic regression were used to examine the characteristics and predictors of stress-coping types. Out of all the women undergoing infertility treatment who completed a self-report survey, 30.8% had a weak mixed coping type, 35.9% had a strong mixed coping type, and 33.3% had a passive coping type. The strong mixed treatment type was compared to weak mixed treatment type, with the following results: infertility adaptation (OR = 17.71, *p* < 0.000), spousal support (OR = 4.50, *p* = 0.021), infertility counseling experience (OR = 7.14, *p* = 0.010). Comparing the strong mixed coping type with the passive coping type, resilience (OR = 9.11, *p* < 0.000) was shown. It is necessary to strengthen resilience and provide a receptive attitude and spousal support to women undergoing infertility adaptation to help them relieve stress and develop functional coping.

## 1. Introduction

In 2020, the total fertility rate in Korea was 0.84; it decreased from the previous year (0.92) to a state of the lowest fertility [1]. However, the infertility rate is increasing by approximately 5% each year [2]. Although policies providing support to combat infertility are being implemented to address this issue, the psychosocial needs of women undergoing infertility treatment need to be addressed, as the experiences of infertility treatment can lead to emotional maladjustment [3].

Infertility refers to the inability to become pregnant in one year while living with a spouse and having a normal sexual relationship or the inability to give birth to a viable child [4]. Most women with infertility experience multidimensional suffering, including psychological and physical burdens [5]. Infertility typically has a greater emotional effect on women than men [6], and it is recognized as a factor that causes substantial stress [7]. Furthermore, the psychological and physical burdens associated with infertility treatment are reportedly severe, often leading to discontinuity of treatment [8]. Moreover, stressful experiences can yield negative emotions such as anger, sadness, and guilt, which may lead to disturbances in implantation or miscarriage, thereby reducing the chances of pregnancy [9]. Negative attitudes toward infertility are associated with an increased use of maladaptive coping strategies [10]. Using an appropriate coping strategy can mitigate the psychological burden of infertility and stress during treatment [10]. Therefore, it is highly important for women with infertility to understand the stress of navigating treatment and related problems, as well as develop strategies to utilize their individual resilience [11]. Hence, there is a need to identify the ways that women cope with the stress of infertility and establish a strategy for each type of coping method to enable women to cope functionally with their stress during infertility treatment.

Resilience, a positive coping resource for psychological stress among women with infertility [12] is characterized by high levels of self-esteem, self-efficacy, and optimism, and it enables women to cope with stress effectively using problem-solving skills [13]. Resilience has been reported to be a protective factor for women with infertility that helps women maintain physical, psychological, and social health and reduces perceived psychological distress [12]. According to the stress-coping adaptation theory, individuals use cognitive evaluation based on human and environmental factors to adapt through appropriate coping methods when faced with a stressor [14]. Furthermore, interpretations, responses, and vulnerability to stressful situations vary among women, leading to individual differences in emotional and behavioral responses based on how a situation is interpreted [14]. Adaptation to infertility is the degree to which an individual is cognitively, emotionally, and behaviorally able to cope with the possibility of having or not having children (i.e., prepared for either outcome) [15]. Depending on how the individual copes with the stress of infertility, there is potential to induce a change in one’s attitude toward infertility treatment. This can occur by identifying an individual’s level of adaptation relevance to infertility, and by alleviating the negative emotions experienced during the infertility treatment process. Substantial spousal support has been shown to lower stress—both directly and indirectly—and potentially play a protective role, which can help reduce the use of inappropriate coping strategies and improve the quality of life for women [16]. The existing literature on infertility is limited to the linear relationship between stress, resilience, and spousal support; research on adaptation to infertility is scarce. Furthermore, while research on stress has been conducted, research on methods of coping with stress among women with infertility has yet to be conducted. Hence, this study aims to identify “individual differences” within the sub-domains of stress coping (rather than the sub-domains themselves); identify the distinguishing characteristics of each sub-domain pertaining to the outcome of stress coping during infertility through cluster analysis; and validate the differences between the identified sub-domains to serve as preliminary data for developing personal intervention plans.

## 2. Materials and Methods

### 2.1. Study Sample

A cross-sectional questionnaire survey was conducted in Korea. The study participants, who were recruited through convenience sampling, were women diagnosed with infertility who were undergoing infertility treatment at a fertility specialist clinic in G Metropolitan City. The inclusion criteria included the following: women diagnosed with primary or secondary infertility, women currently receiving infertility treatment at a fertility clinic, and women with infertility that understood the study’s purpose and voluntarily agreed to participate. The sample size was determined based on evidence that multivariate analysis requires a sample size of at least five times the estimated parameter. Since cluster analysis is a multivariate analysis technique, the minimum sample size was set to 110 individuals, five times the 22 measurement variables analyzed. To account for the rate of attrition, a questionnaire was distributed to 130 individuals, and responses from 120 individuals were collected for the final analysis.

### 2.2. Measures

#### 2.2.1. Stress Coping in Women with Infertility

The Stress-Coping Scale for Infertility-Women (CSI-W) is a measurement tool developed by Kim and Ko (2020) [17] that is composed of 17 questions pertaining to 3 factors of active coping and 11 questions pertaining to 4 factors of passive coping. The four factors pertaining to active coping include the following: “confrontation”, “self-control”), “social support (spouse)”, and “social support (colleagues and experts)”. The three factors pertaining to passive coping include the following: “distancing”, “escape”, and “avoidance”.

The first factor of active coping, “confrontation,” comprises the process of accepting the diagnosis of infertility, while the second factor, “self-control,” includes the individual’s effort to break away from compulsive behavior caused by infertility. The third and fourth factors, “social support (spouse)” and “social support (colleagues or experts),” consist of questions for overcoming issues pertaining to infertility through the social support acquired from a spouse, colleagues, or experts. Among the passive coping factors, “distancing” comprises non-realistic beliefs that the situation in question did not occur, while “escape” refers to the assumption that infertility does not exist. “Avoidance” involves strategies to minimize and postpone the acceptance of reality, such as diverting one’s attention or displaying hesitation toward treatment owing to fear that the treatment will fail. Each question involved responses on a 4-point Likert scale ranging from “not at all” (1 point) to “strongly agree” (4 points). A higher score for each sub-domain indicated a stronger expression of the domain-specific characteristics. The reliability of the 17 questions on active coping and the 11 questions on passive coping in this study were found to be Cronbach’s α = 0.79 and Cronbach’s α = 0.79, respectively. The Cronbach’s α of each sub-domain was 0.82, 0.72, 0.72, and 0.72 for active coping and 0.79, 0.56, and 0.52 for passive coping.

#### 2.2.2. Resilience

Resilience was measured using the Korean version of the Connor–Davidson Resilience Scale (K-CD-RISC). This version was validated and verified as reliable by Baek et al. (2010) [18]. It was derived from the Connor–Davidson Resilience Scale (CD-RISC) developed by Connor and Davidson (2003) [19]. The scale consists of 25 questions in total, with the response to each question organized on a 4-point Likert scale ranging from “not at all” (1 point) to “strongly agree” (4 points). A higher score indicated greater resilience. The reliability of K-CD-RISC was demonstrated as Cronbach’s α = 0.93, while the reliability in this study was found to be Cronbach’s α = 0.92.

#### 2.2.3. Fertility Adaptation

A translated and modified version of the Fertility Adjustment Scale (FAS) developed by Glover et al. (1999) [15] was used to measure the degree of adaptation to infertility, particularly pertaining to acceptance of the diagnosis of infertility, treatment, and treatment-related events. The scale consisted of 12 questions on a 5-point Likert scale ranging from “not at all” (1 point) to “strongly agree” (5 points), with a higher score indicating a higher degree of acceptance of infertility. At the time of development, the reliability of the tool was measured as Cronbach’s α = 0.85, while the reliability in this study was found to be Cronbach’s α = 0.73.

#### 2.2.4. Spousal Support

Spousal support was measured using a 23-question instrument developed by Nam (1987) [20], which was modified to suit women with infertility by Park (2007) [21]. Each question was answered on a 5-point Likert scale ranging from “not at all” (1 point) to “always” (5 points). In Park (2007), Cronbach’s α = 0.95, while in this study, Cronbach’s α = 0.93.

### 2.3. Data Collection

Data were collected with approval from the director and nursing department of a fertility clinic in G Metropolitan City. The researcher was in direct contact with women with infertility who had visited the clinic for an outpatient infertility-related treatment between 16 August and 25 September 2021. Informed consent was obtained from all participants. After gaining their consent, the researcher personally distributed the questionnaire to the participants and collected them immediately following completion. Each participant received a small gift for completing the questionnaire.

### 2.4. Statistical Analysis

Data analysis involved a descriptive analysis of the mean and standard deviation of the participants’ general and infertility-related characteristics using the SPSS 25.0 statistical program. Cluster analysis was performed on the types of stress coping in infertility using the K-means non-hierarchical classification method, which minimizes the difference between the distance and median between groups. The differences in various variables for each type of stress coping for infertility stress were analyzed using the *χ*^2^ test, Fisher’s exact test, one-way ANOVA, and Schéffe test. The predictive factors for each type of stress coping were identified through multinomial logistic regression analysis.

### 2.5. Ethical Considerations

This study was conducted following the approval of the University Bioethics Review Board (assignment number 2-1041055-AB-N-01-2021-37). The survey was only made available to those who understood and agreed to the study’s purpose after reading the recruitment document outlining the details related to the purpose of the study, the study period, recruitment, participant conditions, research procedure, and confidentiality. The document also stated that participants were free to withdraw at any time during the study without penalty, and that the collected data would not be used for purposes other than the purposes of this study, which would be protected in accordance with the Personal Information Protection Act. Lastly, the document contained information on the encryption and statistical processing of data to prevent external exposure. It also informed participants their data would be disposed after the completion of the study.

## 3. Results

### 3.1. Participant Characteristics

#### 3.1.1. General Characteristics

The age distribution of the participants was as follows: 40 participants (33.4%) were in the 31–35 age group, 36 participants (30.0%) were in the over-41 age group, and 33 participants (27.5%) were in the 36–40 age group. Additionally, 64 participants were non-religious (53.3%), 108 participants (90.0%) had an educational level of university diploma or higher, and 73 participants (60.8%) were employed. The participants’ years of marriage ranged from under three years (48 participants, 40.0%), 3–5 years (37 participants; 30.9%), and 5 years or longer (35 participants; 29.1%). Regarding income, 41 participants (34.2%) had a monthly income of under 2 million won, while 38 participants (31.6%) had a monthly income between 2–3 million won. Additionally, 93 participants (77.5%) only had a spouse, while 22 participants (18.3%) had a spouse and children (Table 1). 

#### 3.1.2. Infertility-Related Characteristics

The causes of infertility were provided by the participants as follows: unknown (44 participants; 36.7%), female reproductive issues (36 participants; 30.0%), both male and female reproductive issues (26 participants, 21.6%), or male reproductive issues (14 participants, 11.7%). The general duration of the treatment period for the majority of participants was under 3 years (70 participants; 58.3%), followed by 3–5 years (28 participants; 23.3%). The methods of infertility treatment used by participants included in vitro fertilization (IVF) (88 participants; 73.3%), induced ovulation (17 participants; 14.2%), and artificial insemination (12 participants; 10.0%). A total of 50 participants (41.7%) had never undergone IVF, while 21 participants (17.5%) had undergone 4–6 rounds of IVF. A total of 64 participants (53.3%) had never had a miscarriage, while 34 (28.3%) had experienced a natural miscarriage. Most participants (81 participants; 67.5%) had a proactive attitude toward infertility treatment, while 38 participants (31.7%) were moderately proactive. The greatest challenges caused by infertility were identified by participants as being psychological (76 participants; 63.3%) and health related (14 participants; 11.7%). In total, 82 participants (68.4%) had previously received consultation for infertility (OB-GYN), while 33 participants (27.5%) had no history of infertility consultations (Table 1).

### 3.2. Levels of Stress Coping, Resilience, Infertility Adaptation, and Spousal Support

The average scores of the sub-domains of active stress coping, within the context of infertility, were 3.19 ± 0.38 points for confrontation, 2.93 ± 0.54 points for self-control, 3.18 ± 0.60 points for social support (spouse), and 2.26 ± 0.67 points for social support (peers or experts). The average scores of the sub-domains of passive stress coping were 3.17 ± 0.66 points for distancing, 3.44 ± 0.60 points for escape, and 2.80 ± 0.64 points for avoidance. The average score for resilience was 3.45 ± 0.59 points, for adaptation to infertility was 3.74 ± 0.52 points, and for spousal support was 4.10 ± 0.55 points (Table 2).

### 3.3. Types of Stress Coping

Based on the severity of stress-coping data gathered in the study, three clusters were identified as most prevalent and were analyzed using K-means cluster analysis. [Cluster 1] was organized into active coping: confrontation (3.14 points), self-control (2.70 points), social support (spouse) (3.10 points), and social support (peers/expert) (2.18); and passive coping: distancing (2.46 points), escape (2.95 points), and avoidance (2.37 points). [Cluster 2] was organized into active coping: confrontation (3.33 points), self-control (3.41 points), social support (spouse) (3.59 points), and social support (peers/expert) (2.71); and passive coping: distancing (3.54 points), escape (3.62 points), and avoidance (2.91 points). [Cluster 3] was organized into active coping: confrontation (3.10 points), self-control (2.63 points), social support (spouse) (2.83 points), and social support (peers/expert) (1.84); and passive coping: distancing (3.42 points), escape (3.71 points), and avoidance (3.08 points).

All sub-domains showed significant differences between clusters. The cluster analysis on the sub-domains of stress coping in infertility revealed similarities between clusters 1 and 2. They had higher scores than cluster 3 in the active coping of confrontation, self-control, and spousal and peer/expert support. A comparison based on average scores revealed that passive coping scores and active coping scores were similarly high. Hence, cluster 2 was classified as the “strong mixed coping type,” which uses a combination of active and passive coping. Although cluster 1 involves the acceptance of infertility, unlike cluster 2, it is characterized by a low level of active coping and it does not involve actively controlling and coping with reality. Hence, cluster 1 can be classified as the “weak mixed coping type,” which uses similar levels of passive and active coping. Cluster 3, however, was classified as the “passive coping type,” in which, unlike the other clusters, low levels of active coping and high levels of passive coping—such as distancing, escape, and avoidance—were observed. The number of participants classified into each stress-coping type were as follows: 37 participants (30.8%) in [Cluster 1: weak mixed coping type]; 43 participants (35.9%) in [Cluster 2: strong mixed coping type]; and 40 participants (33.3%) in [Cluster 3: passive coping type] (Table 3).

### 3.4. Characteristic Research Variables Based on the Types of Stress Coping

Resilience (F = 18.33, *p* < 0.001), adaptation to infertility (F = 18.58, *p* < 0.001), and spousal support (F = 8.82, *p* < 0.001) were all found to be statistically significant stress-coping types. The resilience score was higher in Cluster 2 than in Clusters 1 and 3. The adaptation to infertility scores were higher in Clusters 2 and 3 than in Cluster 1. Spousal support scores were higher in Cluster 2 than in Clusters 1 and 3 (Table 4).

### 3.5. Predicted Characteristics by Type of Stress Coping

In order to predict characteristics specific to each stress-coping method, multinomial logistic regression analysis was performed by using [Cluster 1: weak mixed coping type] and [Cluster 3: passive coping type] as reference groups. When [Cluster 2: strong mixed coping type] was compared with [Cluster 1: weak mixed coping type], significant factors distinguishing the two types were identified. These types of stress-coping factors included adaptation to infertility, spousal support, and previous experience receiving a consultation for infertility. The odds ratio of classification into [Cluster 2: strong mixed coping type] is 17.71 times higher (95% CI: 3.53~88.91, *p* < 0.001) when a woman’s level of adaptation to infertility is high. Meanwhile, high levels of spousal support result in an odds ratio 4.50 times higher (95% CI: 1.25~16.22, *p* = 0.021), and previous experience receiving consultation for infertility results in an odds ratio 7.14 times higher (95% CI: 1.61~31.79, *p* = 0.010). When [Cluster 2: strong mixed coping type] was compared with [Cluster 3: passive coping type], the significant distinguishing factor between the two types was identified as resilience. With higher resilience, the odds ratio of being classified into [Cluster 2: strong mixed coping type] is 9.11 times higher (95% CI: 2.74~30.25, *p* < 0.000) (Table 5).

## 4. Discussion

This study aimed to identify the types and characteristics of coping with infertility-related stress among women with infertility. This discussion will focus on the outcomes of the study. Cluster 1 was labeled as the weak mixed coping type, cluster 2 the strong mixed coping type, and cluster 3 the passive coping type. [Cluster 1] identifies a type of person who uses active coping, albeit to a lesser degree than [Cluster 2], and uses passive coping at a similarly low level in combination with active coping. Hence, [Cluster 1] is referred to as the “weak mixed coping type” (30.8%). As a type that often employs the coping method of denial, [Cluster 1] describes those who rely on their spouse when facing stressful situations or emotions related to infertility and experience difficulty in expressing emotions. They are thus unable to engage in active coping. [Cluster 2] identifies a type that freely mixes active and passive coping, with high scores in the sub-domains of both active and passive coping. [Cluster 2] is referred to as the “strong mixed coping type” (35.9%), and it comprises those who can objectively think about the causes of problems, separating the situation and emotions caused by the stress of infertility to maintain optimism. [Cluster 3] engages in passive coping more than any other cluster and is referred to as the “passive coping type” (33.3%). It describes those who try to escape from the emotions caused by situations and events related to infertility stress.

Among the three types of stress-coping clusters identified in this study, the “weak mixed coping type [Cluster 1]” is characterized by a direct acceptance of infertility, albeit followed by an inability to actively control and cope with the stress and a concurrent lack of passive coping. Those in [Cluster 1] employed confrontation and social support (spouse) more frequently than self-control among the sub-domains of active coping. The characteristics of women with infertility in [Cluster 1], such as older age, longer duration of treatment, or a longer period of infertility following marriage, led to greater feelings of depression [22]. Although the participants’ ages or lengths of treatment were not found to be significantly different across clusters in this study, ages and lengths of treatment were observed to be higher (40.5% aged 41 years, 56.7% receiving treatment for 3–5 years) in [Cluster 1]. Nevertheless, these factors may be revisited in a future study.

Women who display the characteristics of [Cluster 1] find it challenging to admit to having infertility or express the emotional pain caused by infertility to others out of fear of social prejudice and negative views [23]. However, the emotional response to infertility differs as time passes [24]. Following a diagnosis of infertility, women first experience shock, followed by disconnection from their mothers and distrust of the world, anger, criticism, shame, and guilt [25]. In other words, when women struggle with the challenges of infertility, their inability to cope is heightened by their inability to express their emotional pain. Interventions to help women with infertility express their emotions independently and adequately through words and behaviors are necessary. This can be achieved through guidance involving praise and encouragement for active coping and support for passive coping.

As spousal support has been revealed to have a positive effect on partners’ adaptations and emotional stabilities [9], an intervention to improve women’s self-control through spousal support is needed for [Cluster 1], in which women demonstrate low levels of self-control and avoidant tendencies. Participation in programs or counseling for more efficient communication between partners may also encourage positive coping through sharing the pain and emotions of infertility with one’s spouse, an important support system in infertility treatment.

The characteristics of the “strong mixed coping type” [Cluster 2] involve high levels of engagement in both active and passive coping with infertility stress. This group experiences the highest engagement of active coping among the three types of stress coping. [Cluster 2] also demonstrated the use of passive coping such as distancing, escape, and avoidance as means to minimize psychological pain in stressful situations, along with a coping style that involved identifying problems to seek solutions. This suggests that women with infertility seek ways to escape reality upon recognition that they cannot remain stagnant in a hopeless situation, but concurrently become increasingly perplexed with their condition, experiencing feelings of isolation through skepticism and loss [26].

Women with infertility who fall into these “types” can adequately cope with infertility stress using various tailored coping methods. Impatience and anxiety may increase if a woman is repeatedly unable to become pregnant despite ongoing infertility treatment. At this time, a mental appreciation of the use of passive coping as a method of taking a break from emotions and solving problems is needed. Depending on the treatment outcomes, emotional fluctuations and unstable demeanors are possible. Nevertheless, [Cluster 2] demonstrates adequate control and focus on their emotions, prompting continuous monitoring. As women classified as this type may present different coping styles based on the psycho-emotional changes experienced in each stage of the treatment process following diagnosis, interventions are needed to help them utilize active coping through positive feedback.

The “passive coping type” [Cluster 3] can be characterized by low levels of active coping and high levels of passive coping. In this type, “escape” was the coping method most utilized, whereby women did not think of infertility in a realistic manner, instead believing that the problem has not occurred. They therefore engaged in hypothetical thinking that infertility did not exist [17]. Such characteristics can be interpreted as tendencies to expect serendipitous solutions without recognizing the problem as requiring active solution-seeking or escaping the problem altogether. This type uses an avoidant coping style such as escaping experiences, which worsens feelings of depression and heightens stress levels following IVF. This type demonstrates tendencies that increase distress and warrant close attention [23]. As persistent feelings of depression can lead to delays or interruptions in treatment, proactive communication and support are critical during and beyond the planning stages of infertility treatment. Indeed, strategies to understand the psychological challenges faced by women with infertility and apply various techniques to encourage them to employ active coping are much needed. Emotion-focused coping, which is classified as passive coping, may be effective in temporarily controlling emotional pain, but it cannot fundamentally address the cause of stress [27]. In other words, a greater use of passive/avoidant coping weakens problem-solving skills, making it more difficult to identify an appropriate coping strategy to alleviate the problem [26]. Interventions that encourage the development of a positive support system and positive coping strategies and discourage passive or avoidant coping are essential.

Statistically significant differences were observed in the resilience, adaptation to infertility, and spousal support of all types of stress coping in the context of infertility. Resilience was statistically significantly higher in [Cluster 2: strong mixed coping type] than in [Cluster 1: weak mixed coping type] and [Cluster 3: passive coping type]. Resilience was a distinguishing factor between [Cluster 2: strong mixed coping type] (3.83 points)—characterized by an understanding of infertility, solution-oriented active coping, and passive coping to minimize psychological pain—and [Cluster 3: passive coping type] (3.25 points). It is likely that the “strong mixed coping type,” which demonstrates high resilience, considers the negative experience of infertility as part of life even in the event of a failed pregnancy, and regards overcoming the situation as a necessary part of adapting [28]. This supports the findings of Kim and Lee (2019) [29], who indicated a significant correlation between active stress coping and resilience. It is presumed that resilience can act as a resource to overcome the stressful situation of infertility by using one’s strengths. It will be necessary to provide an appropriate intervention to identify factors that inhibit women’s abilities to reinforce resilience independently and induce positive change.

Adaptation to infertility was higher in [Cluster 2: strong mixed coping type] and [Cluster 3: passive coping type] than in [Cluster 1: weak mixed coping type]. The degree of adaptation to infertility, by type of stress coping, was 3.96 points for the “strong mixed coping type” and 3.85 points for the “passive coping type,” both higher than the 3.36 points for the “weak mixed coping type” and thus indicative of greater acceptance of the diagnosis of infertility. Adaptation to infertility requires cognitive, emotional, and behavioral changes [15]. The degree of adaptation varies by individual and it is known to affect an individual’s interpretation and coping ability in stressful situations such as infertility [30]. Such variance may be interpreted as differences in the method of accepting infertility. Hence, women with infertility must be encouraged to plan their lives independently, moving away from a life focused solely on pregnancy through positive reinterpretation and acceptance of infertility in a low-control situation.

Spousal support, by type of stress coping, was higher in the “strong mixed coping type” (4.35 points) than in the “weak mixed coping type” (3.89 points) and “passive coping type” (4.01 points). Positive interactions and communication with their partners decrease active avoidant coping and increase active coping among women with infertility [23]. If their spouse recognizes infertility as a shared problem, women are more reliant on their spouse and able to form a closer relationship compared to before the diagnosis of infertility [22]. Infertility procedures, including artificial insemination and IVF, are difficult without the support and cooperation of one’s partner [21]. Hence, promoting understanding and cooperative attitudes toward a partner who is undergoing infertility treatment is essential, as expectations for partners may be different for each stage of treatment.

The analysis of the differences in general characteristics based on the type of stress coping revealed a statistically significant difference in family structure and burden of treatment costs. Participants who live only with their spouse were more likely to be classified as the “weak mixed coping type” than the “strong mixed coping type” or “passive coping type.” Family support can help women recover and adapt successfully to stressful situations [31]. Nevertheless, the belief that one’s lack of children is a flaw can lead to changes in familial relationships owing to confusion over senses of loss and achievement [32]. Considering that relational factors are as important as personal factors in infertility, family support—a societal relational factor involving family—likely affects women’s qualities of life [31]. Hence, in the infertility process, it is important to consider ways to increase family participation beyond the spouse. Interventions to encourage positive relationships between family members are necessary to heal from the distance or conflict caused by a lack of understanding.

In terms of financial stress, 70.7% [33] and 53.3% [34] of participants responded that the treatment costs for infertility treatment were “burdensome.” Indeed, the high costs of the procedures pose significant economic burdens for those with infertility. It was reported that 70.8% of participants [33] also found treatment costs to be “burdensome,” which supports the findings of this study. From October 2017, the cost of infertility treatment could be covered through health insurance, while in 2019, the coverage was expanded from 130% below the standard median income to 180% below the standard median income. Furthermore, an active response strategy has been under establishment following the implementation of support for costs related to IVF as well as artificial insemination [35]. The need to develop a counseling program for women with infertility to manage stress and strengthen coping strategies is evident, given the physiological, psychological, and social problems related to infertility. Furthermore, repeated studies involving a larger sample may be needed to establish a basis for policy change and implementation.

Among infertility-related characteristics based on stress-coping type, statistically significant differences were seen in previous experiences of patients receiving consultation for infertility. The “strong mixed coping type” had the highest proportion of participants with previous experience receiving consultation for infertility among the three types, at 76.7%. Counseling is highly encouraged in the event of multiple failures or unwanted procedure outcomes, and when the woman is feeling challenged by treatment [36]. Psychosocial interventions must be undertaken meticulously according to the patient’s condition or symptoms. Counseling to encourage early detection—to identify factors that aggravate the situation—and healthy coping behaviors when faced with unexpected consequences are needed to aid women in returning to their normal levels of functioning.

When examining participant characteristics by stress-coping type, a woman’s likelihood to be a “strong mixed coping type,” in comparison to a “weak mixed coping type,” is much higher when adjustment to infertility is high, recognition of spousal support is high, and there is more experience with counseling. The main factor that distinguishes “weak mixed coping” from “strong mixed coping” is adaptation to infertility, which is related to self-stigma and a sense of failure [37] (Sternke & Abrahamson, 2015). Such a tendency makes treatment more difficult and often prolongs infertility [38]. Substantial spousal support, on the other hand, directly and indirectly reduces stress and acts as a potential protective factor, helping reduce the use of inappropriate coping strategies related to infertility and improve quality of life [25]. The average sub-domain score of those in the “weak mixed coping type” is low in self-control and high in spousal support. Indeed, this type relies more heavily on spousal support than on themselves when coping with stress; hence, spousal support is an important factor. Interventions for partners that promote social support, including intermarital infertility-related communication and spousal support, should be developed for couples who are starting infertility treatment.

A woman’s likelihood to be a “strong mixed coping type” rather than a “passive coping type” is higher with higher resilience. Resilience is a necessary component to increase a woman’s capacity to endure and grow from challenges. Hence, it is necessary to mediate the psycho-emotional problems that emerge throughout the stages of infertility treatment through detailed education, guidance, and support, from the planning process to the treatment process. Moreover, a plan to establish positive coping through a program to strengthen resilience should be considered in program development.

Factors related to resilience, adaptation to infertility, and spousal support are important in determining the type of stress coping adopted by a woman with infertility. It has been demonstrated that having a partner who perceives infertility as a shared problem helps to highlight women’s strengths and competencies while they are facing the stresses of infertility and encourages a positive reinterpretation and acceptance of infertility. This partnership plays an important role in determining the woman’s coping type. Therefore, a program that aims to support women’s stress coping in infertility should involve deliberation on the woman’s individual situation; the factors pertaining to the cognitive, emotional, and behavioral aspects of infertility; and the marital relationship.

This study identified the characteristics of women’s stress coping, a variable that is not often considered in the current circumstances of low fertility. Policy to support women with infertility is emerging as an important solution to the increasingly severe problem of low fertility. Furthermore, this study explored the need to constructively and functionally improve the coping methods of women with infertility who face challenges due to inappropriate coping. This study is significant as it presents a classification of the types of stress coping through cluster analysis, a method that overcomes the issues that occur when classifying stress coping using mean or median values. In other words, by identifying the factors that determine the types of stress coping among women with infertility, the findings of this study will contribute to the development of specific intervention strategies for each type of stress coping.

## 5. Conclusions

This study classified the types of stress coping among women with infertility and confirmed the influence of resilience, adaptation to infertility, and spousal support in determining their type of coping. The need to establish a support system to help women find active coping strategies is clear. Given that methods of coping worsen and change throughout their exposure to repeated stress caused by the various procedures and tests in the early stages of diagnosis, reviewing the negative emotions that may arise at each step of the treatment process may be helpful. As there are limitations in generalizing the findings of this study, a repeated study involving a larger sample size from multiple clinics and various treatment methods, ages, and types of infertility (primary, secondary, etc.) is suggested. Second, this study only involved women, which suggests the need for a follow-up study on infertile couples that accounts for the increasing number of males with infertility. Third, there is a need to identify and add factors influencing the classification of the stress-coping types identified in this study, and develop and validate an infertility counseling intervention program by coping type. Fourth, although stress-coping types were classified through cluster analysis in this study, future studies should involve qualitative interviews with participants for a more in-depth exploration of the characteristics of each type.

## Figures and Tables

**Table 1:** General and infertility characteristics (*N* = 120).

General Characteristics	Categories	n (%)	Infertility Characteristics	Categories	n (%)
Age (years)	20~25	1 (0.8)	Infertility factors	Unexplained	44 (36.7)
	26~30	10 (8.3)		male	14 (11.7)
	31~35	40 (33.4)		female	36 (30.0)
	36~40	33 (27.5)		both	26 (21.6)
	≥41	36 (30.0)	Infertility treat period (years)	1–<3	70 (58.3)
Religion	Yes	56 (46.7)		3–< 5	28 (23.3)
	No	64 (53.3)		5–<10	17 (14.2)
Education level	≤High school	12 (10.0)		≥10	5 (4.2)
	≥College	108 (90.0)	Types of infertility treatment	IUI	12 (10.0)
Job	Yes	73 (60.8)		IVF	88 (73.3)
	No	47 (39.2)		OI	17 (14.2)
Marriage period (years)	1~<3	48 (40.0)		Etc	3 (2.5)
	3~<5	37 (30.9)	IVF experience	No	50 (41.7)
	5~<7	19 (15.8)		Once	18 (15.0)
	≥7	16 (13.3)		2–3	18 (15.0)
Monthly income (10,000 won)	<200	41 (34.2)		4–6	21 (17.5)
	200~<300	38 (31.6)		6	4 (3.3)
	300~<500	32 (26.7)		≥7	9 (7.5)
	≥500	9 (7.5)	Abortion experience	No	64 (53.3)
Family member	Spouse	93 (77.5)		S.A	34 (28.3)
	Spouse and child	22 (18.3)		A.A	22 (18.4)
	parents-in-law	5 (4.2)	Attitude to treat infertility	Active	81 (67.5)
				Moderate	38 (31.7)
				Passive	1 (0.8)
			Difficulty with infertility	Economic difficulties	12 (10.0)
				Difficulties in marital relationships	3 (2.5)
				Psychological problems	76 (63.3)
				Family relations	2 (1.7)
				Health problems	14 (11.7)
				Social prejudice	7 (5.8)
				etc.	6 (5.0)
			Experience of counseling for infertility	None	33 (27.5)
				Yes (Doctor)	82 (68.4)
				Yes (Nurse)	1 (0.8)
				Yes (etc.)	4 (3.3)

Notes: IVF, In Vitro Fertilization; IUI, Intrauterine Insemination; OI, Ovulation Induction; SA, Spontaneous Abortion; AA, Artificial Abortion.

**Table 2:** Coping with infertility stress, resilience, infertility adaptation, and spouse support (*N* = 120).

		Factors	M ± SD	Range
Stress coping	Active	Confrontation	3.19 ± 0.38	1~4
		Self-control	2.93 ± 0.54	1~4
		Social support (spouse)	3.18 ± 0.60	1~4
		Social support (colleague or expert)	2.26 ± 0.67	1~4
	Passive	Distancing	3.17 ± 0.66	1~4
		Escape	3.44 ± 0.60	1~4
		Avoidance	2.80 ± 0.64	1~4
Resilience			3.45 ± 0.59	1~5
Infertility adaptation			3.74 ± 0.52	1~5
Spousal support			4.10 ± 0.55	1~5

Notes: M, Mean; SD, Standard Deviation.

**Table 3:** Infertility stress-coping type (*N* = 120).

Factors		Type	Cluster1 ^a^	Cluster2 ^b^	Cluster3 ^c^
		n (%)	37 (30.8)	43 (35.9)	40 (33.3)
Active coping	Confrontation	M ± SD	3.14 ± 0.33	3.33 ± 0.39	3.10 ± 0.38
		F(*p*)	4.89 (0.009)		
		Scheffé	c, a < b		
	Self-control	M ± SD	2.70 ± 0.39	3.41 ± 0.40	2.63 ± 0.42
		F(*p*)	47.20 (<0.001)		
		Scheffé	c, a < b		
	Social support (spouse)	M ± SD	3.10 ± 0.41	3.59 ± 0.42	2.83 ± 0.64
		F(*p*)	24.57 (<0.001)		
		Scheffé	c, a < b		
	Social support (colleague or expert)	M ± SD	2.18 ± 0.53	2.71 ± 0.58	1.84 ± 0.64
		F(*p*)	25.05 (<0.001)		
		Scheffé	c < a < b		
Passive coping	Distancing	M ± SD	2.46 ± 0.47	3.54 ± 0.44	3.42 ± 0.45
		F(*p*)	65.49 (<0.001)		
		Scheffé	a < c, b		
	Escape	M ± SD	2.95 ± 0.60	3.62 ± 0.48	3.71 ± 0.41
		F(*p*)	26.89 (<0.001)		
		Scheffé	a < b, c		
	Avoidance	M ± SD	2.37 ± 0.55	2.91 ± 0.57	3.08 ± 0.59
		F(*p*)	16.13 (<0.001)		
		Scheffé	a < b, c		

Notes: M, Mean; SD, Standard Deviation. ^a^ = weak mixed coping type. ^b^ = strong mixed coping type. ^c^ = passive coping type.

**Table 4:** Differences in variables according to the type of infertility stress coping (*N* = 120).

	Categories	Type	Cluster 1	Cluster 2	Cluster 3
		n (%)	37 (30.8)	43 (35.9)	40 (33.3)
General characteristics	Family member	Spouse	35 (92.1)	27 (73.0)	31 (68.9)
		Child and Parents-in-law	3 (7.9)	10 (27.0)	14 (31.1)
		*x*^2^ (*p*)	9.22 (0.010)		
	Treatment cost Affordability ^∥^	Hard	32 (86.5)	25 (58.1)	28 (70.0)
		Medium	3 (8.1)	10 (23.3)	10 (25.0)
		Easily	2 (5.4)	8 (18.6)	2 (5.0)
		*x*^2^ (*p*)	10.67 (0.034)		
Infertility characteristics	Counseling ^∥^ experience.	None	15 (40.5)	6 (14.0)	12 (30.0)
		Yes (Doctor)	22 (59.5)	33 (76.7)	27 (67.5)
		Yes (Nurse)	0 (0.0)	0 (0.0)	1 (2.5)
		Yes (etc.)	0 (0.0)	4 (9.3)	0 (0.0)
		*x*^2^ (*p*)	15.29 (0.011)		
Resilience		M ± SD	3.22 ± 0.53	3.83 ± 0.54	3.25 ± 0.49
		F(*p*)		18.33 (<0.001)	
		Scheffé		a, c < b	
Infertility adaptation		M ± SD	3.36 ± 0.43	3.96 ± 0.47	3.85 ± 0.47
		F(*p*)		18.58 (<0.001)	
		Scheffé		a < c, b	
Spousal support		M ± SD	3.89 ± 0.46	4.35 ± 0.48	4.01 ± 0.59
		F(*p*)		8.82 (<0.001)	
		Scheffé		a, c < b	

Notes: M, Mean; SD, Standard Deviation. ^∥^ = Fisher’s exact test.

**Table 5:** Predicted characteristics by type of stress coping (*N* = 120).

Reference Group		Categories	Cluster2 ^††^					
			*B*	SE	OR	*p*	95% CI	
Cluster 1 ^†^		(constant)	−20.34	4.29		0.000		
	Resilience		0.77	0.67	2.17	0.245	0.59 -	7.98
	Infertility adaptation		2.87	0.82	17.71	0.000	3.53 -	88.91
	Spousal support		1.50	0.65	4.50	0.021	1.25 -	16.22
	Family member							
		Spouse	(Reference)					
		Child and parents-in-law	1.94	0.99	6.99	0.050	1.00 -	48.92
	Treatment cost affordability							
		None	(Reference)					
		Hard	−0.99	0.74	0.37	0.184	0.09 -	1.60
	Counseling experience							
		No	(Reference)					
		Yes	1.97	0.76	7.14	0.010	1.61 -	31.79
Cluster3 ^§^		(constant)	−10.50	3.48		0.003		
	Resilience		2.21	0.61	9.11	0.000	2.74 -	30.25
	Infertility adaptation		−0.14	0.61	0.87	0.818	0.26 -	2.88
	Spousal support		0.57	0.57	1.76	0.323	0.57 -	5.43
	Family member							
		Spouse	(Reference)					
		Child and parents-in-law	−0.52	0.62	0.59	0.402	0.17 -	2.01
	Treatment cost affordability							
		None	(Reference)					
		Hard	0.10	0.56	1.10	0.861	0.37 -	3.27
	Counseling experience							
		No	(Reference)					
		Yes	1.29	0.69	3.63	0.062	0.94 -	14.08
	Likelihood Ratio		*x*^2^ = 179.042, *p* < 0.001					
	Cox and Snell *R*^2^		0.498					
	Nagelkerke *R*^2^		0.561					

Notes: SE, Standard Error; OR, Odds Ratio; CI, Confidence Interval. ^†^ = weak mixed coping type, ^††^ = Strong mixed coping type. ^§^ = Passive coping type.

## Data Availability

Data are available by request to the corresponding author.
